# Effect of Low-Frequency Alternating Magnetic Field-Assisted Liquid Fermentation on Extracellular Polysaccharides Structural Characteristics and Biosynthesis of *Pleurotus citrinopileatus*

**DOI:** 10.3390/foods15162794

**Published:** 2026-08-10

**Authors:** Jingya Qian, Dazhou Lu, Feng Wang, Shuhao Huo, Bin Zou, Haile Ma

**Affiliations:** 1 School of Food Science and Engineering, Jiangsu University, 301 Xuefu Road, Zhenjiang 212013, China; 2 Institute of Food Physical Processing, Jiangsu University, 301 Xuefu Road, Zhenjiang 212013, China

**Keywords:** low-frequency alternating magnetic field, *Pleurotus citrinopileatus*, extracellular polysaccharides, polysaccharide composition, transcriptome

## Abstract

Low-frequency alternating magnetic field (LF-AMF) was applied to liquid fermentation of *Pleurotus citrinopileatus* for the production of extracellular polysaccharides (EPS). Two types of EPS (POL1 and POL2) were secreted by *P. citrinopileatus* under normal fermentation conditions, while only one type of EPS (POL3) was produced under LF-AMF-assisted fermentation. The study demonstrated that LF-AMF-assisted fermentation altered the monosaccharide composition, molar ratios of component monosaccharides, and molecular weight distribution of EPS. LF-AMF enhanced the flocculation activity of EPS in a concentration-dependent and dosage-specific manner. Transcriptomic analysis revealed a total of 32,413 differentially expressed genes (DEGs) (|log2(fold-change)| ≥ 1, *p* < 0.05) between *P. citrinopileatus* under LF-AMF-assisted fermentation and normal fermentation. Of those, 5818 genes were up-regulated and 26,595 genes were down-regulated. The DEGs were enriched in metabolic pathways and biosynthesis of secondary metabolite. Genes involved in glycolysis and gluconeogenesis, such as hexokinase, 6-phosphofructokinase, fructose-1,6-phosphate aldolase, glyceraldehyde 3-phosphate dehydrogenase and glucose-1,6-diphosphatase were up-regulated. Additionally, genes in the tricarboxylic acid (TCA) cycle, including citrate synthase, isocitrate dehydrogenase and α-ketoglutarate dehydrogenase were also up-regulated. These findings suggest that LF-AMF-induced transcriptional alterations in glycolysis may modulate the metabolic supply of precursors for EPS biosynthesis, thereby affecting the biosynthetic process and altering the compositional characteristics of EPS.

## 1. Introduction

*Pleurotus citrinopileatu* (*P. citrinopileatus*)*,* a member of *Basidiomycotina,* is one of the valuable medicinal and edible fungi native to East and Southeast of Asia. It has been reported that *P. citrinopileatus* contains a variety of bioactive compounds, including polysaccharides, dietary fiber, proteins, amino acids, trace elements, and various minerals [1,2]. These bioactive compounds are traditionally extracted from field-cultivated fruiting bodies of *P. citrinopileatus*, with well-characterized structural properties and diverse biological activities [3]. However, field cultivation is limited by its long cultivation cycle and the difficulty in controlling environment conditions. For industrial-scale production of edible fungi, the liquid fermentation was employed as a preferable method. Liquid fermentation can produce *P. citrinopileatus* mycelia and fermentation broth in a short time with controlled fermentation conditions.

Polysaccharides, the important ingredients of *P. citrinopileatus* have a variety of functional properties, including antioxidant, anti-inflammatory, hepatoprotective and immunomodulatory [4,5,6,7]. Those polysaccharides can be sourced from broth mycelia and fermentation broth. Intracellular polysaccharides (IPS) derived from mycelium and extracellular polysaccharides (EPS) obtained from fermentation broth have similar bioactivities to those derived from fruiting bodies. The yield and structural properties of EPS are influenced by the fermentation medium and cultivation conditions. For example, the type of carbon and nitrogen sources, together with pH affected EPS yield from *P. citrinopileatus* [8]. The compositions and molecular weight (MW) of EPS produced by *P. citrinopileatu* were also affected by nitrogen sources [9]. The structural characteristics of polysaccharides, namely monosaccharide composition, MW, functional groups, and glycosidic linkage types, are correlated with their biological activities [10,11,12].

EPS has been applied in food and health-care product formulations, functioning as a natural texturant, stabilizer, and delivery vehicle [13,14]. However, the relatively low yield of EPS limits its application in food and other industries. Researchers have been focused on the development of novel and efficient EPS production strategies in recent years. Yang et al. [15] optimized the fermentation conditions by employing a genetic algorithm combined with an artificial neural network to increase exopolysaccharide production by *Pediococcus pentosaceus*. Accumulating studies have demonstrated that specific physical field treatments can promote mycelial growth, increase biomass accumulation, and enhance the biosynthesis of active metabolites in fungal fermentation systems. Such green non-invasive microbial regulation strategies have been widely validated to effectively optimize fermentation product profiles [16], providing a reference for applying magnetic field regulation to edible fungal polysaccharide biosynthesis. Magnetic field, as a non-thermal technology, has potential for improving the microbial growth and fermentation efficiency. Static magnetic field enhanced methane production via stimulating the growth and composition of microbial community [17], while low intensity alternating magnetic field increased the yield of polysaccharides during the submerged fermentation of *Grifola frondosa* [18] and *Antrodia camphorata* [19]. However, the effect of magnetic field on the chemical structure and biosynthesis of fermentation metabolites have not been reported.

Due to advancement of omics technology, the global synthetic metabolic network of various organisms is extensively explored. In recent years, transcriptome has been employed for elucidating the biosynthesis of microbial metabolites, such as exopolysaccharide. Transcriptomic analysis revealed that nitrogen sources modulated EPS biosynthesis in *Streptococcus thermophilus* IMAU20561 by regulating multiple metabolic routes, including amino acid biosynthesis, glycolysis, the phosphotransferase system, and fructose/mannose metabolism [20]. Carbohydrate metabolism was involved in exopolysaccharide biosynthesis by *Monascus purpureus* at different fermentation times [21].

In this manuscript, low-frequency alternating magnetic field (LF-AMF) was applied to *P. citrinopileatus* liquid fermentation for the production of EPS. After the fermentation, EPS was extracted and purified to investigate the chemical structure. Furthermore, transcriptomic analysis was used to explore the molecular mechanisms underlying EPS biosynthesis in *P. citrinopileatus* under LF-AMF condition.

## 2. Materials and Methods

### 2.1. Materials and Reagents

*P. citrinopileatus* CICC 14010 was obtained from China Center of Industrial Culture Collection (Beijing, China) and preserved in College of Food and Biological Engineering, Jiangsu University (Zhenjiang, China). D-Glucose, D-Galactose, D-Xylose, L-Rhamnose, D-Mannose and L-Arabinose were purchased from Sigma Chemical Co. (St. Louis, MO, USA). Reagents used in spectral analysis were of chromatographic grade. All other reagents were of analytical grade.

### 2.2. Fungus Culture and Fermentation

*P. citrinopileatus* was activated and cultured on Potato Dextrose Agar (PDA) medium, which was also used as seed culture medium. After cultured at 28 °C with shaking at 150 r/min for 7 days, the seed culture was inoculated into fermentation mediums containing glucose (35 g/L), yeast powder (5 g/L), MgSO4·7H_2_O (0.75 g/L) and KH_2_PO_4_ (1.5 g/L) at an inoculate rate of 10% (*v*/*v*). Fermentation was carried out in 250 mL Erlenmeyer flasks with a working volume of 100 mL. The initial pH of the fermentation medium was adjusted to 6.0. All fermentation trials were performed at 28.3 °C with shaking at 149 r/min for 6.2 days.

For LF-AMF-assisted fermentation, the initial intervention time was 24 h after the inoculation, with the treatment time of 6 h and the intensity of 40 Gs. The LF-AMF was generated by a custom-built low-intensity alternating magnetic field shaker system, powered by a 220 V AC supply and regulated by a TDGC2-3k (3000 VA) single-phase contact voltage regulator. Each shaker well was wound with identical coil turns (compatible with 250 mL Erlenmeyer flasks) to ensure uniform magnetic flux density across parallel samples. The field intensity was adjusted via the regulator and calibrated with a gaussmeter before each experiment. The 50 Hz sine-wave field had a uniformity of ±5% in the central sample area, with its direction perpendicular to the liquid surface. The fermentation temperature was maintained at 28.3 ± 0.5 °C throughout exposure, and the no-field control was incubated in an identical shaker at the same temperature without magnetic field exposure.

### 2.3. Extraction and Purification of Exopolysaccharide from Fermented Broth of P. citrinopileatus

After the fermentation, mycelia and the fermented broth were separated by filtration. The collected broth was added with ethanol (1:4, *v*/*v*), stored at 4 °C overnight, and then centrifuged at 8000 r/min for 10 min to obtain the precipitate. The precipitate was redissolved in distilled water, deproteinized by trichloroacetic acid (TCA) precipitation, dialyed by dialysis bag and lyophilized as described by Wang et al. [22]. In brief, 16% trichloroacetic acid was added to the mixture, left at 4 °C overnight, and centrifuged at 8000 r/min for 30 min to obtain supernatant. After the protein precipitate was discarded, pre-cooled 95% ethanol was added to the supernatant, stored at 4 °C for 8 h, and centrifuged to obtain the EPS precipitate. The precipitate was solubilized with 10 mL of water and the crude polysaccharide solution was dialyzed in 8000–14,000 Da dialysis bags for 4 days. The crude polysaccharide (C-EPS) was then lyophilized for later use.

C-EPS (5 mg) was dissolved in 5 mL of distilled water. The resulting solution was then loaded onto a DEAE-52 anion-exchange column and eluted with distilled water, followed by NaCl solutions at 0.1, 0.2, and 0.3 mol/L. Eluates were collected in tubes (10 mL/tube) at a flow rate of 1 mL/min for each gradient, and the polysaccharide content was measured using phenol-sulfuric acid method. A Sephadex G-150 gel column was employed for further purification, with distilled water serving as the mobile phase. Fractions of 1.5 mL were collected at a flow rate of 0.3 mL/min, and the eluate was monitored using the phenol-sulfuric acid method. The purified EPS (P-EPS) was obtained after isolation and purification.

### 2.4. EPS Characterization

#### 2.4.1. UV Spectroscopy Analysis

P-EPS (1 mg/mL) was detected with an ultraviolet-visible spectrometer (UV 5200, Aoxi Instrument Co., Ltd., Shanghai, China) and scanned in a range of 190–400 nm [23].

#### 2.4.2. FT-IR Spectroscopy Analysis

P-EPS (1 mg) was mixed with dry KBr (150 mg) and tableted. The configurations of monosaccharide were scanned with a FT-IR spectrometer (Nicolet 380, Thermo Fisher Scientific, Waltham, MA, USA) in a range of 4000–400 cm^−1^ [24].

#### 2.4.3. Monosaccharide Compositions Analysis

P-EPS (5.0 mg) was hydrolyzed with trifluoroacetic acid (TFA) (3.0 mL, 2.0 mol/L) at 105 °C for 6 h. The solution was evaporated to dryness to obtain polysaccharide hydrolysate. The hydrolyzed sample was acetylated by adding hydroxylamine hydrochloride (10.0 mg), inositol hexaacetate (2.0 mg), acetic anhydride (0.5 mL) and pyridine (0.5 mL) and reacting at 90 °C for 30 min. Seven monosaccharide standards (D-Glucose, D-fructose, D-Xylose, D-Galactose, L-Rhamnose, D-Mannose and L-Arabinose) (2.0 mg) were also acetylated similarly. The products were dissolved in dichloromethane, and detected by gas chromatography (GC) (7890A, Agilent Technologies, Inc., Santa Clara, CA, USA) equipped with an HP-5 ms column (30 m × 0.32 mm × 0.25 μm) and a flame ionization detector (FID). GC conditions were set as described by Wang et al. [25]. The inlet and detector were set to 280 °C and 300 °C, respectively. The column temperature was held at 130 °C for 5 min before being raised to 240 °C at 4 °C/min. Nitrogen, hydrogen, and air were supplied at flow rates of 37, 30, and 400 mL/min, respectively, with a split ratio of 40:1.

#### 2.4.4. Molecular Weight Determination

The Mw of P-EPS was determined by high performance gel-permeation chromatography (HPGPC) (Agilent-1200, Agilent Technologies Co., Ltd., Santa Clara, CA, USA) equipped with a gel column (7.8 mm × 300 mm) (TSK-GEL G4000 PWXL, Tokyo, Japan) and a refractive index detector (RID). The concentration of P-EPS was 1 mg/mL, and the sample solution was filtered through a 0.45 µm membrane before injection. Chromatographic conditions were as follows: injection volume, 200 µL; mobile phase, 0.1 mol/L NaCl; flow rate, 0.5 mL/min; column temperature, 25 °C.

#### 2.4.5. Congo Red Assay

The triple helix structure of P-EPS was determined by the Congo red method [26]. P-EPS Solution (1 mg/mL), Congo red reagent (100 μmol/mL), and NaOH (1.0 mol/L) were prepared, respectively. P-EPS Solution (1 mL) was mixed with Congo red reagent (2 mL), and then NaOH solution (1 mol/L) were added to the mixed solution to achieve the required NaOH concentrations (0~0.5 mol/L). After incubating at room temperature for 10 min, the maximum absorption wavelength of the mixture was measured by an ultraviolet-visible spectrophotometer in the range of 400 nm–600 nm.

#### 2.4.6. Thermal Characteristics Analysis

Thermogravimetric analysis (TGA) was performed using a thermal gravimetric analyzer (TG-DTA 6200SII, SII NanoTechnology Inc., Chiba, Japan). Lyophilized P-EPS was placed in a crucible and heated from 0 to 600 °C at a linear heating rate of 10 °C/min in an air atmosphere at a flow rate of 100 mL/min. Differential scanning calorimetry (DSC) was conducted using a DSC 3 (Mettler Toledo, Zurich, Switzerland). The lyophilized P-EPS (5 mg) was placed in an aluminum pan and heated from 0 to 400 °C at a linear heating rate of 10 °C/min in a nitrogen atmosphere.

#### 2.4.7. Emulsifying Activity

The emulsifying activity of C-EPS was determined following the method described by Sran et al. [27] with some modification. C-EPS was prepared at the concentration of 1 mg/mL. A mixture of 1 mL of C-EPS and 0.5 mL of hexadecane was homogenized for 2 min. The absorbance of the mixture was measured at 540 nm after standing at the room temperature for a defined time (0, 30, 60, 90 min, respectively). Emulsifying activity was calculated as follows:

Emulsifying activity (%) = A_t_/A_0_ × 100%.

where At is the absorbance of the defined time, and A_0_ is the absorbance of the initial time.

#### 2.4.8. Flocculation Activity

The flocculation activity of C-EPS was determined based on the method described by Wang et al. [25] with some modification. Firstly, 10 mL of activated carbon suspension solution (5 g/L) was mixed with 0.1 mL of calcium chloride solution (6.8 mmol/L). Then, EPS (0.2–1 mg/mL) was added to the mixture. After standing at 25 °C for 10 min, the supernatant was obtained and its absorbance was measured at 550 nm. The flocculation activity (%) was calculated as follows:

Flocculation activity (%) = (B − A)/B × 100%

where A is the absorbance of the sample suspension, and B is the absorbance of the control.

### 2.5. Transcriptomic Analysis

#### 2.5.1. RNA Isolation and Sequencing

Mycelia from fermentation with and without LF-AMF were collected, washed with sterile water, quickly frozen in liquid nitrogen and stored in refrigerator at −80 °C. Three independent biological replicates from separate fermentation batches were prepared for each group. Total RNA was extracted using Trizol reagent. The quality of RNA was assessed using a Nanodrop spectrophotometer (Thermo Fisher Scientific, Waltham, MA, USA) and the integrity was examined with the RNA Nano 6000 Kit (Agilent Technologies, Inc., Santa Clara, CA, USA). High-quality RNA samples were selected for sequencing library construction, which was performed on the NovaSeq 6000 platform.

#### 2.5.2. Sequencing Data Processing

High-quality clean reads were obtained by removing reads with adapters, low quality, and those with more than 10% N base from the raw data. Then, clean reads were RNA de novo assembled using Trinity software (v2.14.0) and aligned. The expression level of each transcript was calculated according to the reads per kilobase of exon per million mapped reads (RPKM) method.

#### 2.5.3. Functional Analysis of Differentially Expressed Genes (DEGs)

After quality control, clean reads were first aligned to the reference genome for gene quantification. Unmapped reads were assembled de novo to identify novel unannotated transcripts. Differential expression analysis was performed using DESeq2 (v1.34.0) based on raw read counts, with normalization via the median-of-ratios method and multiple testing correction by the Benjamini–Hochberg method. Genes with |log2 fold change| ≥ 1 and adjusted *p*-value (FDR) < 0.05 were defined as differentially expressed genes. DEGs were subjected to Gene Ontology (GO) functional annotation and Kyoto Encyclopedia of Genes and Genomes (KEGG) pathway enrichment analyses.

### 2.6. Data Analysis

All experiments were performed with three independent biological replicates, each sample was analyzed in triplicate as technical replicates, with an exact sample size *n* = 3 (biological replicates) for all assays. Normality of data was verified by the Shapiro–Wilk test, and homogeneity of variance was examined by Levene’s test. One-way ANOVA was applied for single-factor comparisons. For experiments with two factors, two-way ANOVA was used to analyze the main effects of each factor and their interaction effect. Tukey’s HSD test was adopted as the post hoc test for multiple comparisons. Different lowercase letters in the figures indicate statistically significant differences (*p* < 0.05). All statistical analyses were performed using SPSS 22.0 (IBM Corp., Armonk, NY, USA).

## 3. Results and Discussion

### 3.1. Purification of EPS from P. citrinopileatus

**Figure 1:**
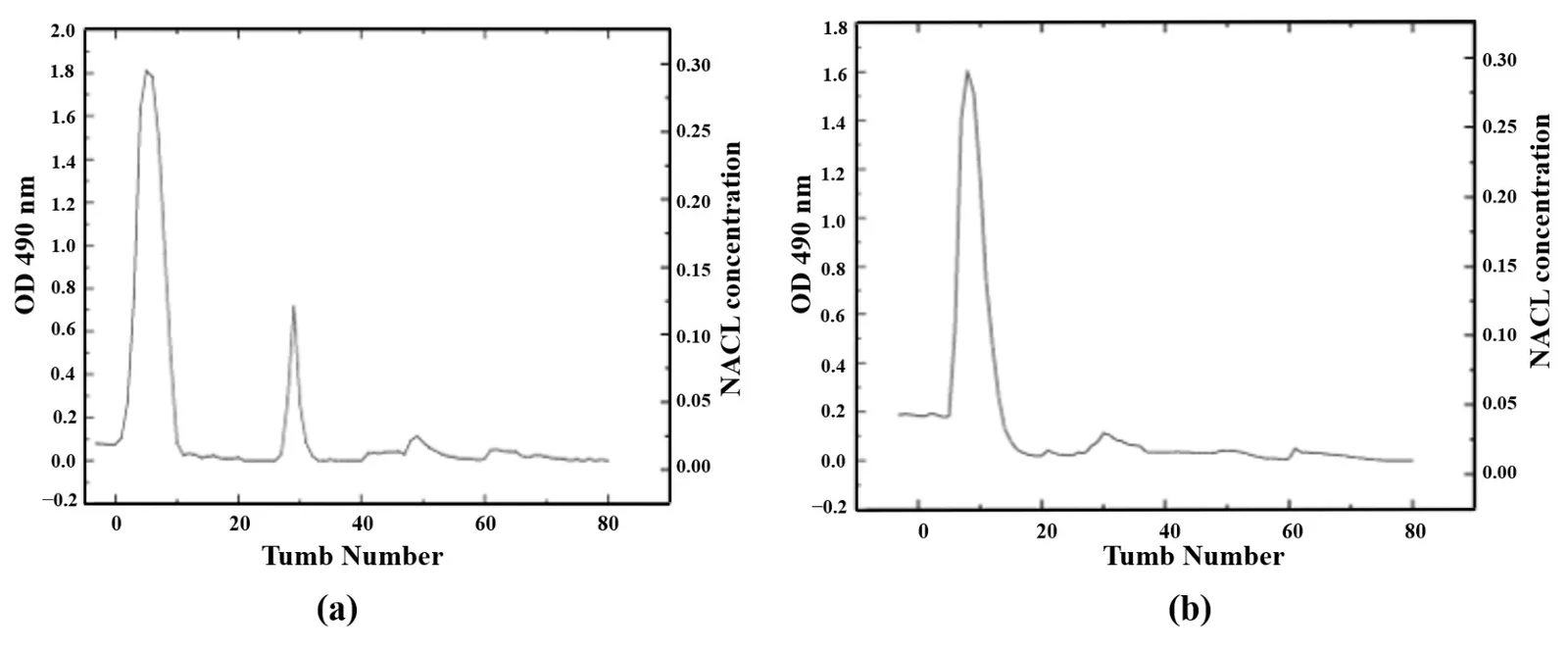
Purification of EPS from *P. citrinopileatus* fermentation unassisted (**a**) and assisted (**b**) by LF-AMF.

The crude EPS (C-EPS) was extracted from the fermented broth of *P. citrinopileatus* and further purified with a DEAE-cellulose column. The elution curves of EPS from *P. citrinopileatus* fermentation unassisted and assisted by LF-AMF are depicted in Figure 1a and Figure 1b, respectively. Normal fermentation provided two types of EPS (POL1 and POL2), while LF-AMF-assisted fermentation only presented one type of EPS (POL3). Therefore, LF-AMF-assisted fermentation altered the types of EPS in the fermented broth of *P. citrinopileatus*.

### 3.2. Structure Analysis of EPS

#### 3.2.1. Analysis of UV Spectrum

**Figure 2:**
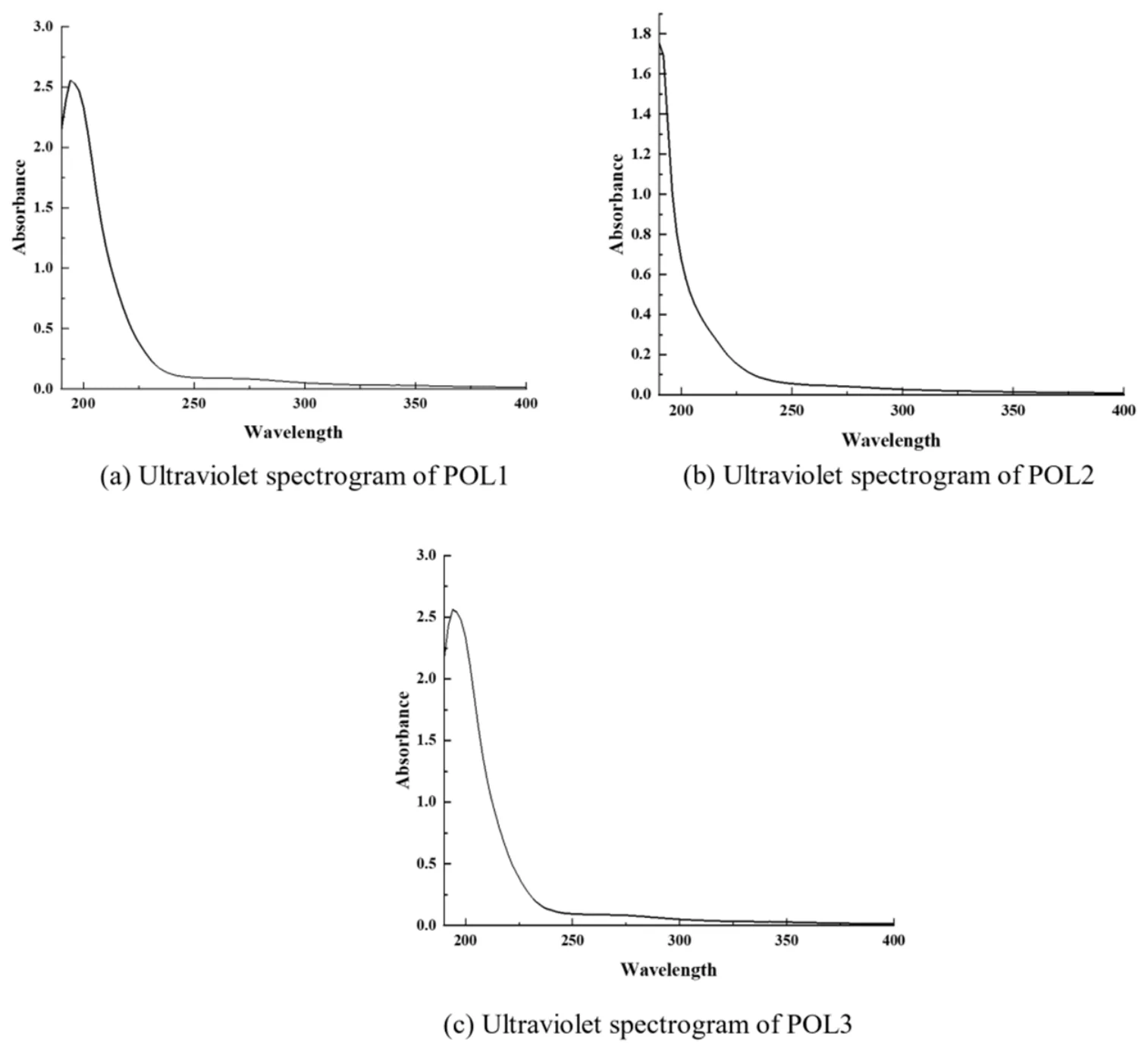
Ultraviolet spectrogram of EPSs (POL1, POL2 and POL3) from *P. citrinopileatus* fermentation.

The UV spectra of the three purified EPSs (POL1, POL2 and POL3) are shown in Figure 2a–c. There were no UV absorption peaks at 260 nm and 280 nm, indicating that POL1, POL2 and POL3 were free of nucleic acids and proteins.

#### 3.2.2. Analysis of FT-IR Spectrum

**Figure 3:**
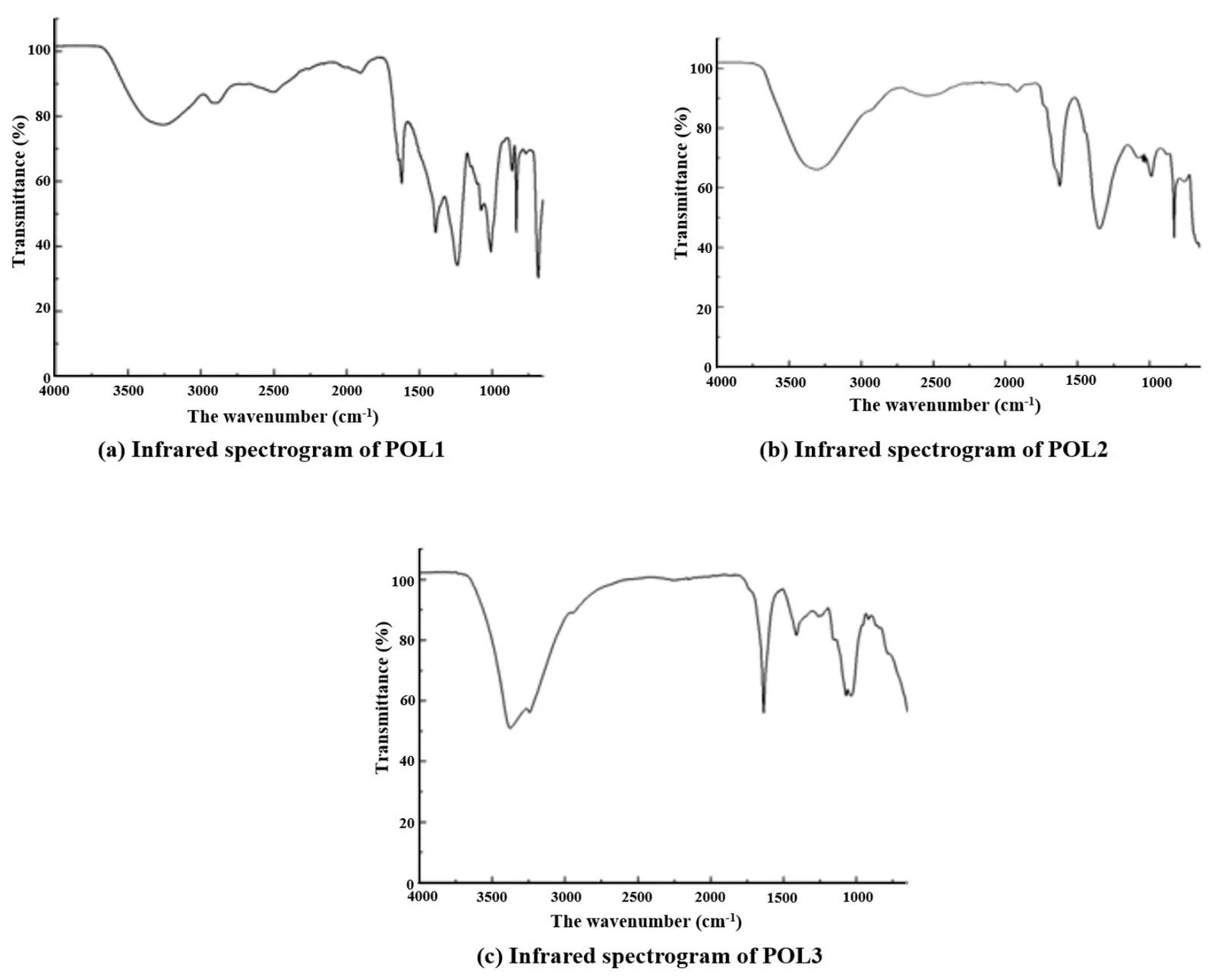
Infrared spectrogram of EPS (POL1, POL2 and POL3) from *P. citrinopileatus* fermentation.

The organic functional groups in POL1, POL2 and POL3 were identified by FT-IR spectroscopy and the results are presented in Figure 3. There strong absorption peaks at 3385 cm^−1^ (POL3), 3315 cm^−1^ (POL1), and 3305 cm^−1^ (POL2) correspond to the stretching vibration of -OH [28]. The weak absorbance peaks at 2943 cm^−1^ (POL3), 2925 cm^−1^ (POL1), and 2919 cm^−1^ (POL2) are attributed to C–H stretching vibrations [29]. The strong absorption peak at 1637 cm^−1^ indicates the stretching vibration of C = O [30]. The absorption peaks at 1637 cm^−1^ (POL3), 1624 cm^−1^ (POL1) and 1627 cm^−1^ (POL2) resulted from the presence of C = O bond. The absorption peak at 1420 cm^−1^ relates to C-H variable angle vibration [31]. POL1, POL2 and POL3 exhibited the absorption peaks of C-H bond bending vibration at 1392 cm^−1^,1356 cm^−1^ and 1414 cm^−1^, respectively. The absorption peaks at 1254 cm^−1^ (POL3) and at 1242 cm^−1^ (POL1) implied the presence of sulfate group. In the range of 1200 to 1000 cm^−1^, the absorption peaks are associated with the stretching vibration of C-O-C and C-O-H [32], indicating that POL1, POL2 and POL3 all contained pyranose ring structure. The absorbance bands at 850 and 931 cm^−1^ propose α-and β-glycosidic bonds [33]. The absorption peaks at 915 cm^−1^ (POL1) and 913 cm^−1^ (POL3) indicated the presence of β-D-glucopyranose. POL1 and POL3 also had absorption peaks at 855 cm^−1^ and 860 cm^−1^, suggesting the existence of an α-glycosidic bond. The absorption peak of POL2 at 832 cm^−1^ also demonstrated the presence of α-glycosidic bond. Based on the results of infrared spectroscopy, it could be inferred that POL1, POL2 and POL3 contain typical polysaccharide functional groups and were classified as pyranose.

#### 3.2.3. Analysis of Monosaccharide Composition of EPS

The biological activities of polysaccharides are governed by their structural attributes, including monosaccharide makeup, molecular weight profiles, and branching extent [34,35].

**Figure 4:**
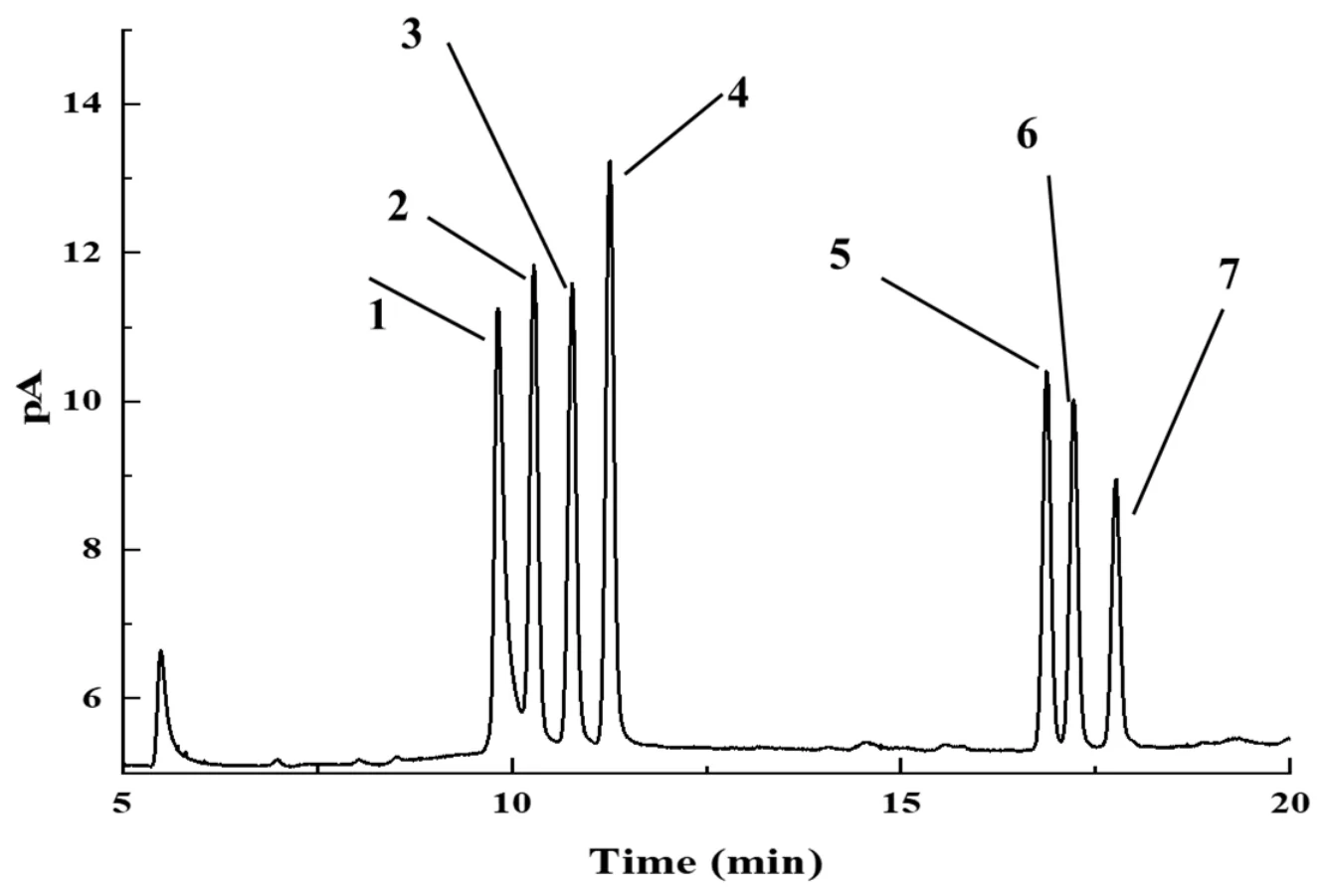
Gas chromatogram of standard monosaccharide. 1: fructose; 2: rhamnose; 3: arabinose; 4: xylose; 5: mannose; 6: glucose; 7: galactose.

**Figure 5:**
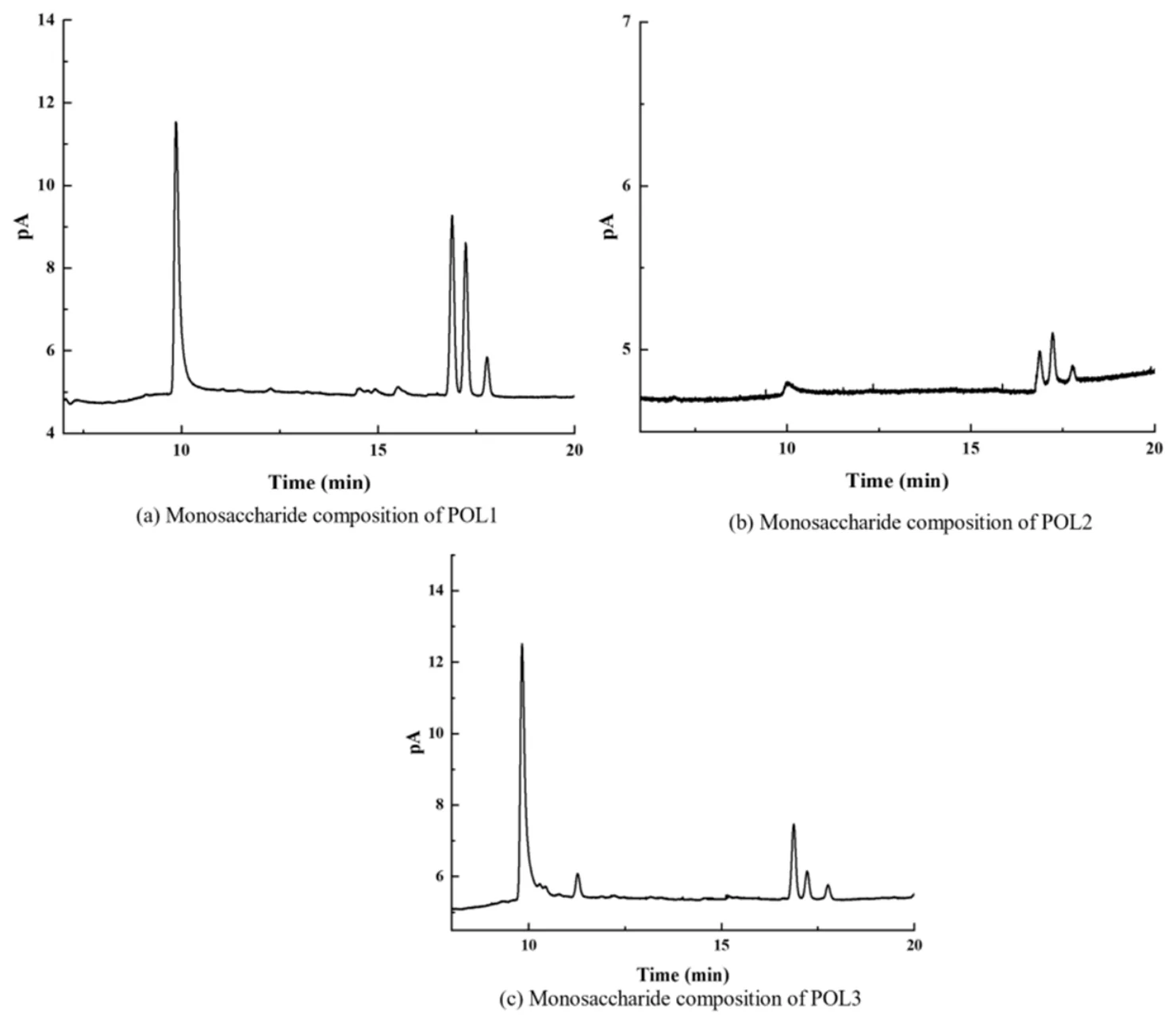
Gas chromatogram of monosaccharide composition of EPSs (POL1, POL2 and POL3) from *P. citrinopileatus* fermentation.

The monosaccharide profiles of POL1, POL2, and POL3, as determined by gas chromatography, are illustrated in Figure 4 and Figure 5, respectively. POL1 was found to comprise fructose, mannose, glucose, and galactose at a relative molar ratio of 4.72:2.81:2.21:0.41. POL2 contained rhamnose, mannose, glucose, and galactose in the proportion of 1.33:1.95:2.33:1.27. POL3 was characterized by the presence of fructose, xylose, mannose, glucose, and galactose with a molar ratio of 21.58:1.96:5.87:1.96:0.56. After LF-AMF-assisted fermentation, the monosaccharide composition of EPS from *P. citrinopileatus* was changed.

#### 3.2.4. Analysis of Molecular Weight of EPS

**Figure 6:**
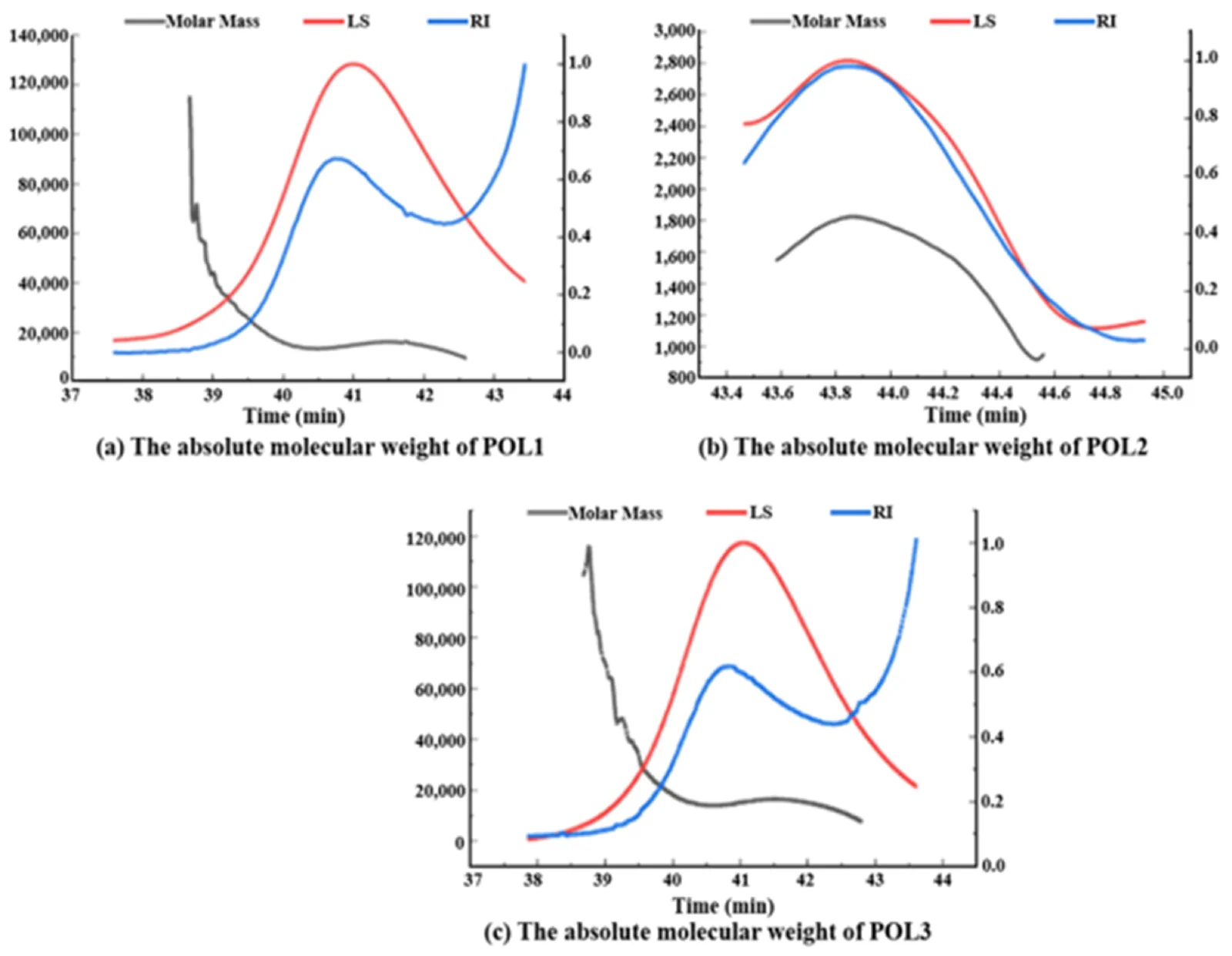
The relative molecular weight of EPSs (POL1, POL2 and POL3) from *P. citrinopileatus* fermentation.

As shown in Figure 6, the relative molecular weights of POL3, POL2 and POL1 were 1.456 × 10^4^ Da, 2.003 × 10^3^ Da and 1.439 × 10^4^ Da, respectively. Molecular weight is a key structural property associated with polysaccharide functional activity. POL3 had the largest molecular weight among the three fractions. LF-AMF-assisted fermentation increased the molecular weight of EPS, a structural feature that may relate to the functional characteristics of the polysaccharide.

#### 3.2.5. Analysis of Triple Helix Conformation of EPS

**Figure 7:**
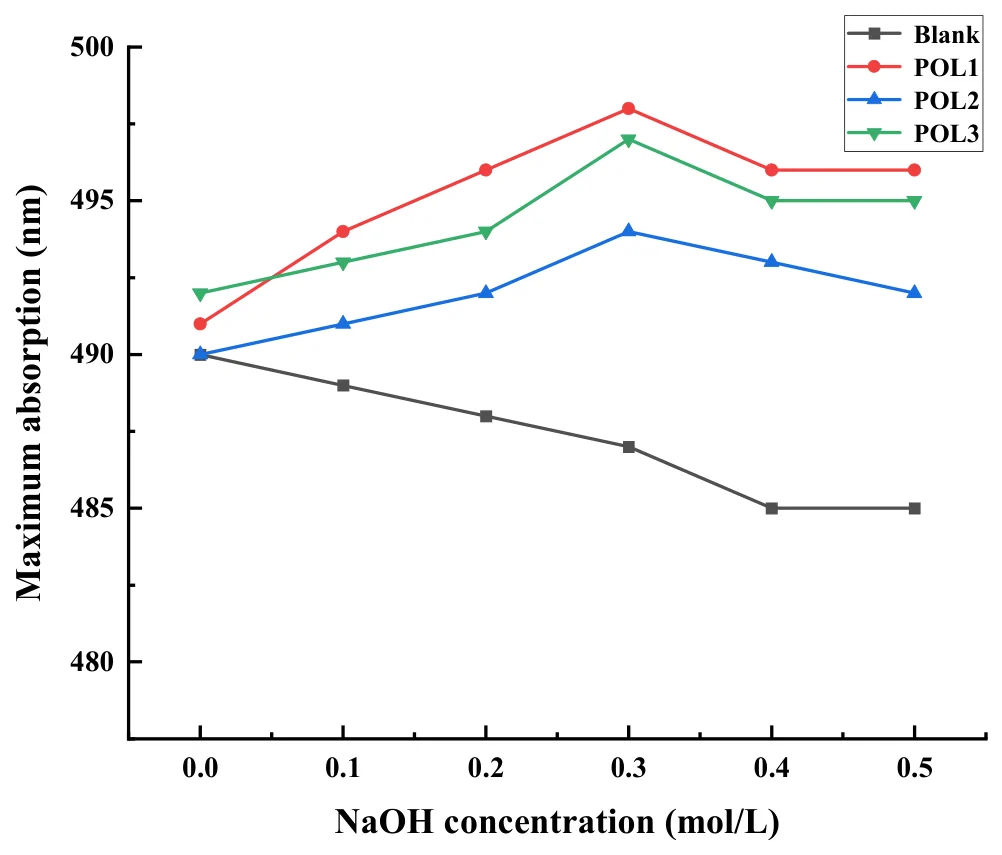
Congo red experimental analysis of EPSs (POL1, POL2 and POL3) from *P. citrinopileatus* fermentation.

Congo red can react with polysaccharides that have a triple helix structure to form a complex in alkaline solution. The triple helix conformation of polysaccharides is confirmed by a red shift in the maximum absorption wavelength of the complex [36]. The Congo red experimental results for POL1, POL2 and POL3 are shown in Figure 7. The absorption wavelengths of POL1, POL2 and POL3 complexes increased with the increase in the concentration of NaOH from 0.1 to 0.3 mol/L. When the concentration of NaOH reached 0.3 mol/L, the maximum absorption wavelengths appeared, indicating the presence of triple helix conformation in these three EPSs. However, the maximum absorption decreased as the concentration of NaOH continued to increase, suggesting that triple-helix conformation of POL1, POL2 and POL3 were disrupted. Polysaccharides with triple helix structures generally exhibit higher bioactivity than those without such conformation [37].

### 3.3. Functional Properties Analysis of EPS

#### 3.3.1. Analysis of Thermal Characteristics of EPS

**Figure 8:**
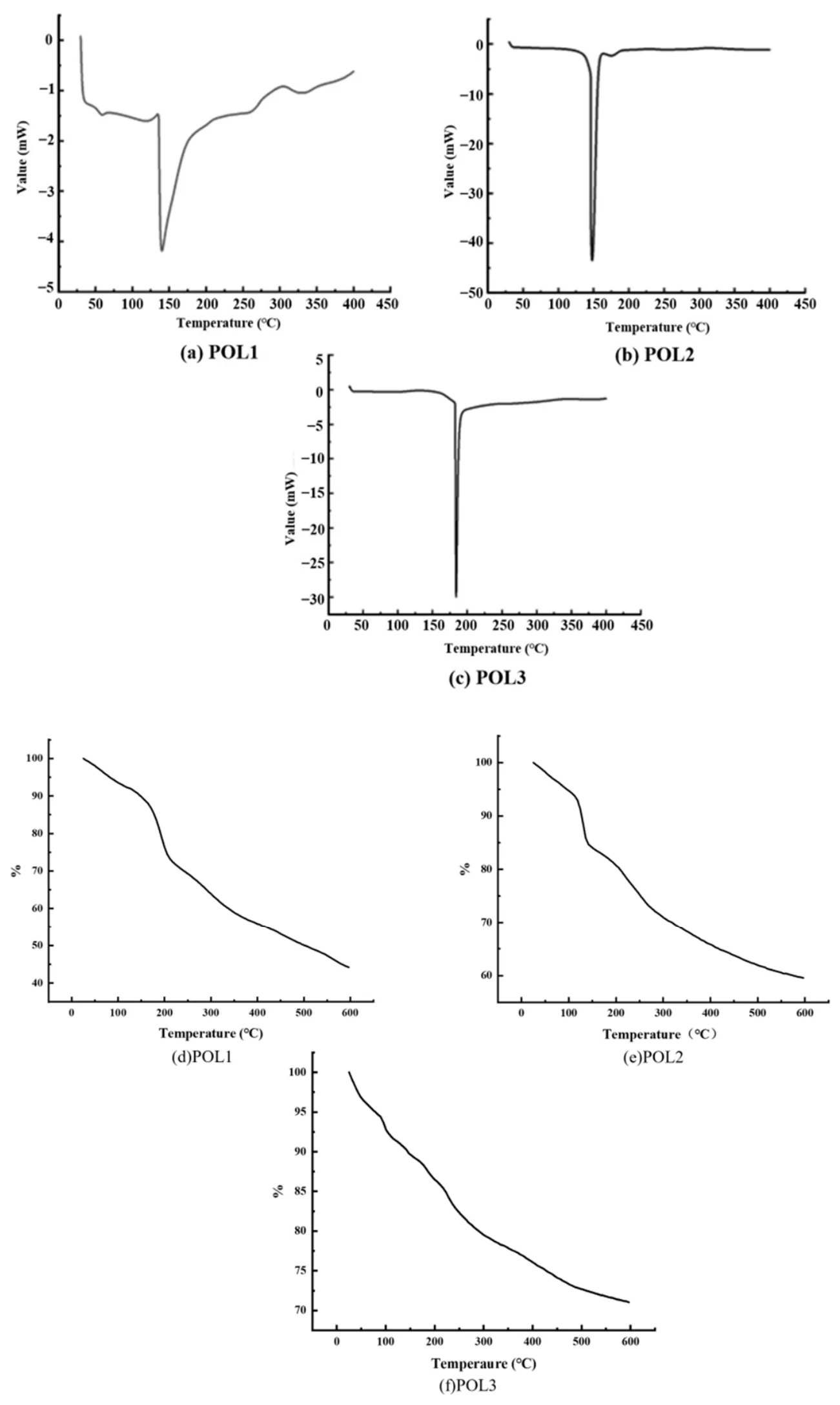
Scanning calorimeter curves of EPSs (POL1, POL2 and POL3) (**a**–**c**) and thermogravimetric analysis curves of EPSs (POL1, POL2 and POL3) (**d**–**f**) from *P. citrinopileatus* fermentation.

The DSC curves of POL1, POL2 and POL3 are shown in Figure 8a–c. All three EPSs displayed an endothermic peak, with transitions occurring in the temperature range of 30–200 °C, attributed to the loss of free and bound water. Among the three EPSs, POL3 had the highest endothermic peak transition temperature, indicating its superior water binding ability.

The TGA curves of POL1, POL2 and POL3 are presented in Figure 8d–f. All three EPSs showed a three-stage weight loss mode. The initial weight loss of these three EPSs occurred in the temperature range of 30–100 °C, which was mainly due to the water evaporation. The structure of the polysaccharide depolymerizes at about 100 °C, resulting in a decrease in weight [38]. POL3 exhibited a lower weight loss rate (approximately 30% of the original weight) compared to POL1 and POL2, suggesting that thermal stability of EPS was enhanced after LF-AMF-assisted fermentation.

#### 3.3.2. Analysis of Emulsifying Activity

**Figure 9:**
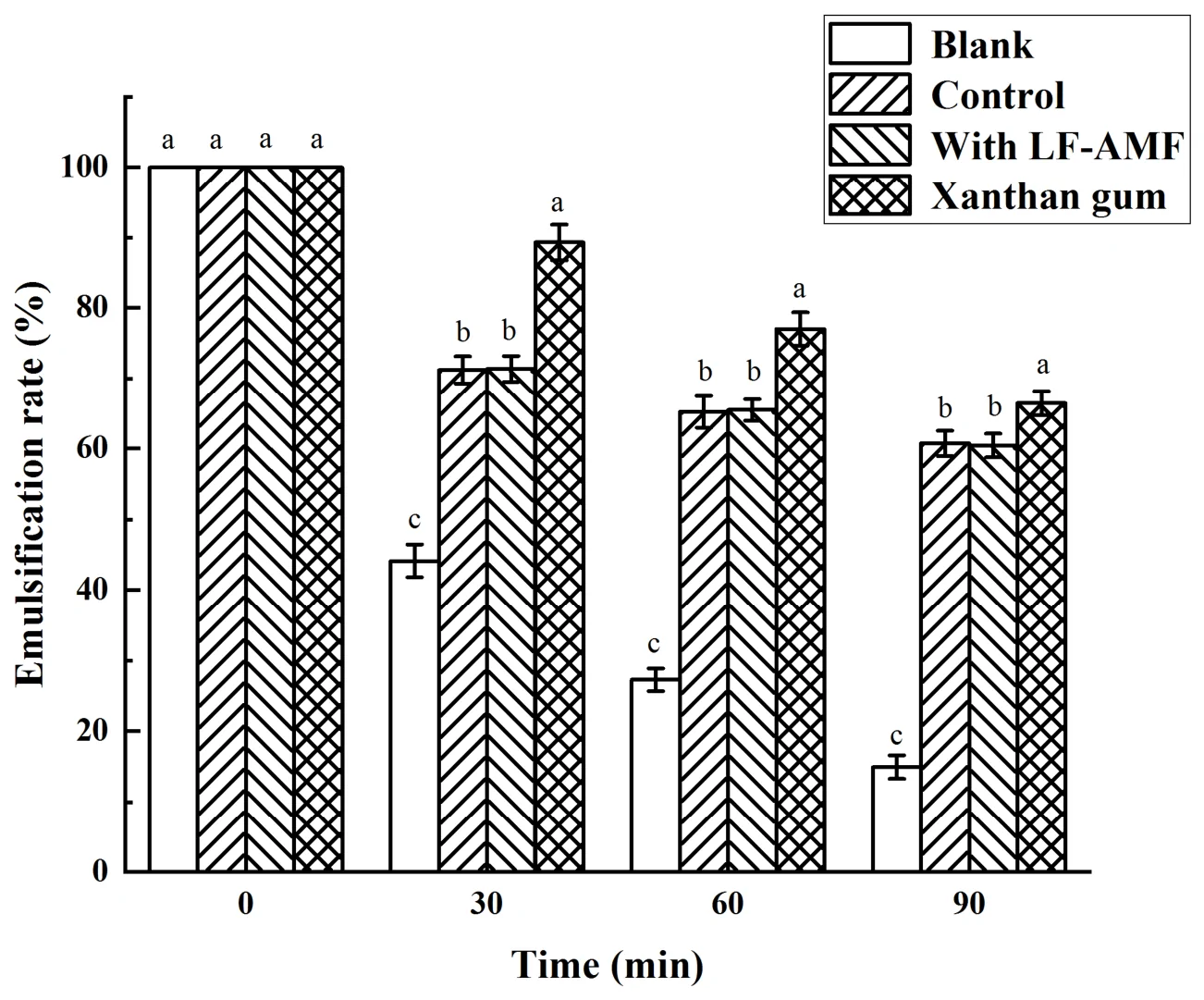
Emulsifying activity of EPSs from *P. citrinopileatus* fermentation unassisted and assisted by LF-AMF. Different lowercase letters indicate significant differences among groups at the same time point (*p* < 0.05).

The emulsifying activity of EPS can be assessed by the duration for which it maintains emulsion stability. The emulsifying activity of EPSs from LF-AMF-assisted and normal fermentation is shown in Figure 9. After standing for 30, 60, and 90 min, the emulsifying activity of EPS from LF-AMF-assisted fermentation was 71.35%, 65.44%, and 60.44%, respectively. The emulsifying activity of EPS from LF-AMF- unassisted fermentation was 71.21%, 65.23%, and 60.74% after standing for 30, 60, and 90 min, respectively. Although the emulsifying activity of EPS from both groups was slightly lower than that of xanthan gum, their emulsifying stability was still satisfactory, indicating potential for broad applications in the food industry.

#### 3.3.3. Analysis of Flocculation Activity

**Figure 10:**
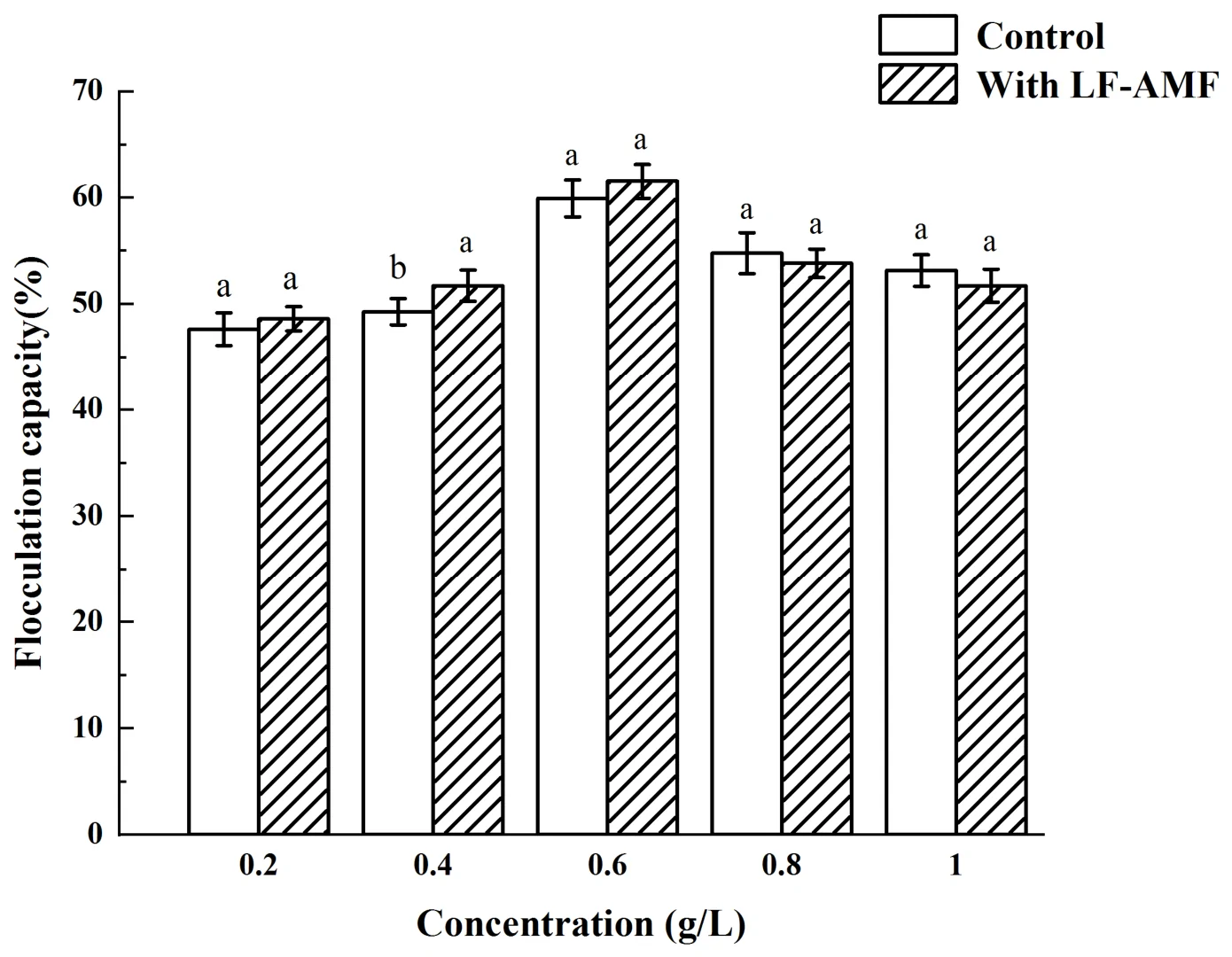
Flocculation activity of EPS from *P. citrinopileatus* fermentation unassisted and assisted by LF-AMF. Different lowercase letters indicate significant differences among groups at the same time point (*p* < 0.05).

The flocculation activity of EPSs from LF-AMF-assisted and normal fermentation is shown in Figure 10. The flocculation activity increased with the EPS concentration in the range of 0.2–0.6 mg/mL, but decreased with further increases in concentration. When the concentration was in the range of 0.6–1 mg/mL, the flocculation activity of EPS decreased. This may be due to particle instability. An excessive amount of flocculant cannot be completely dispersed in the suspension, and only the particles around the polysaccharide can be absorbed [39]. At a concentration of 0.6 mg/mL, the flocculation activity of EPSs from LF-AMF-assisted and normal fermentation reached the highest levels, which were 61.53% and 59.90%, respectively. These results provide experimental support for the potential development of this EPS as a natural flocculant.

### 3.4. Transcriptome Analysis

#### 3.4.1. Sequencing Quality Assessment

As shown in Table 1, all cDNA libraries exhibited high sequencing quality overall. The Q30 base accuracy rate of each sample exceeded 92%, and the GC content distribution was within the normal range for fungal transcriptomes with no obvious bias detected. These results confirmed that the sequencing data was reliable and qualified for subsequent differential expression and functional enrichment analysis.

De novo assembly was performed on clean reads that could not be aligned to the reference genome, yielding a total of 54,465 transcripts with a total length of 56,981,293 bp and an N50 value of 1485 bp, which indicated high assembly quality. Furthermore, as shown in Table 2, the mapping rate exceeded 87.21%, confirming that the data were adequate for subsequent annotation analysis.

#### 3.4.2. Screening of DEGs

**Figure 11:**
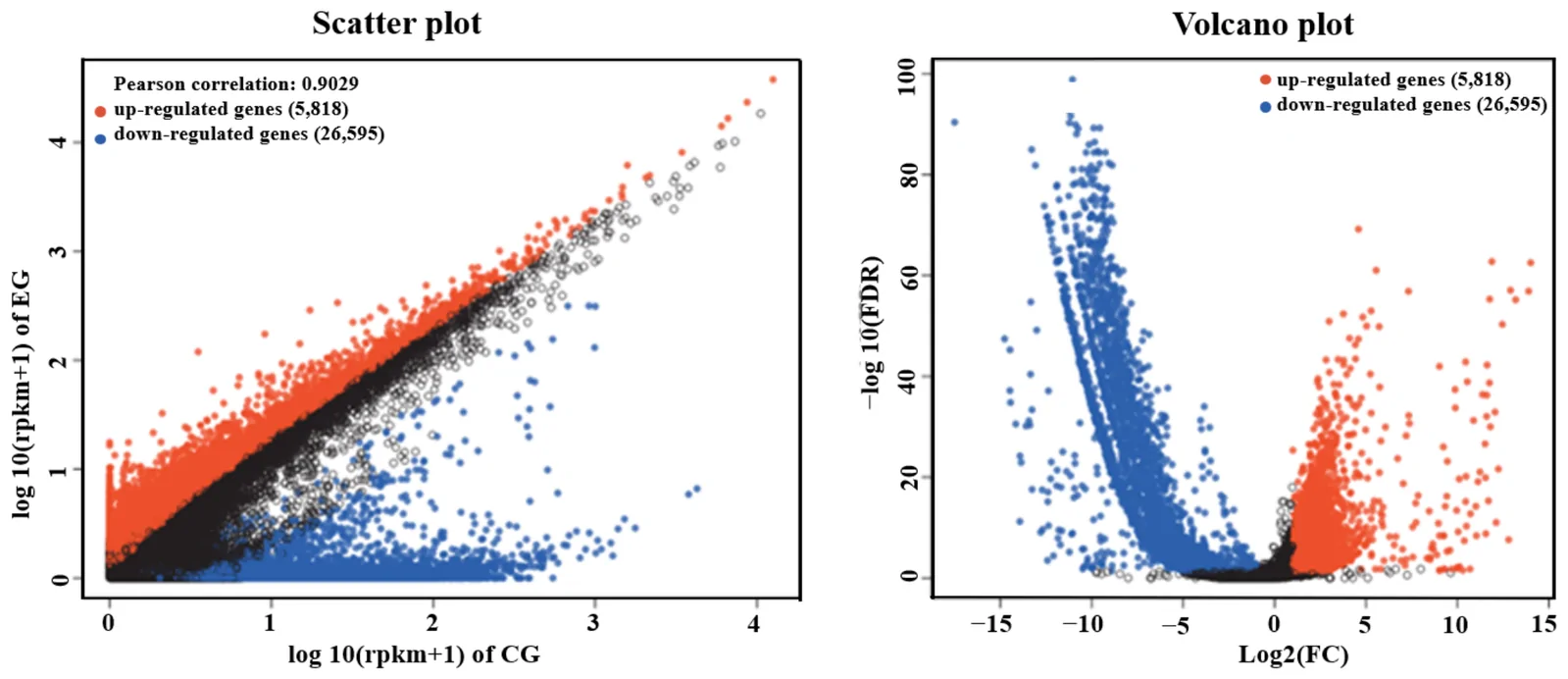
Scatter and volcano plots of DEGs.

A total of 32,413 DEGs (|log2(fold-change)| ≥ 1, *p* < 0.05) were identified between *P. citrinopileatus* under LF-AMF-assisted and normal fermentation. Among these DEGs, 5818 genes were up-regulated and 26,595 genes were down-regulated, revealing a significant alteration in gene expression profiles, with the majority of genes being down-regulated in *P. citrinopileatus* under LF-AMF-assisted fermentation. The volcanic map of the DEGs is presented in Figure 11.

#### 3.4.3. GO Analysis

The Gene Ontology (GO) database is designed to explain gene function and its properties. GO analysis is a method that can associate specific biological functions with DEGs.

**Figure 12:**
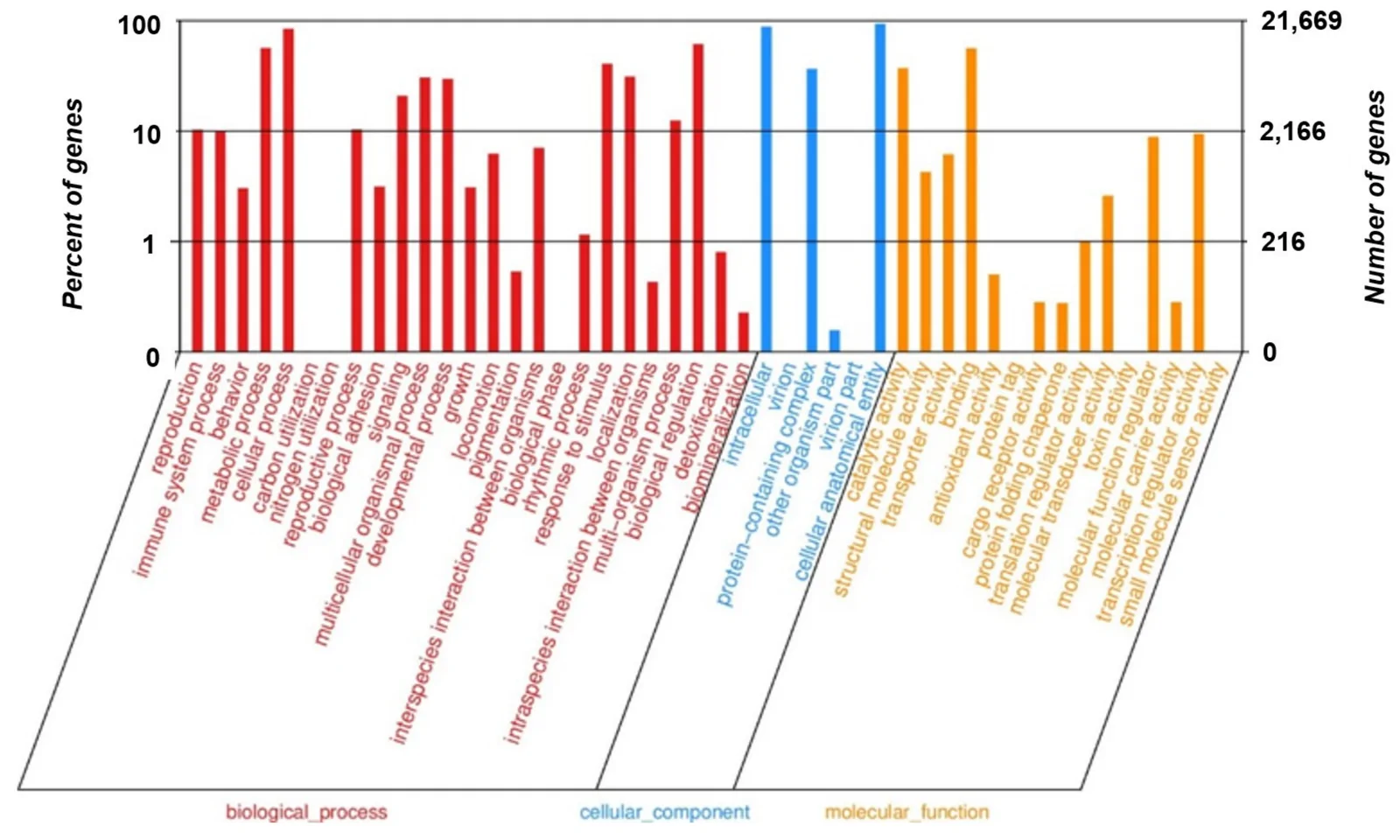
GO annotation of DEG.

As shown in Figure 12, GO annotations were categorized into three major classes, including biological processes, cellular component and molecular function. Biological processes encompassed cellular processes, biological regulation, metabolic processes, etc. Among these, a total of 18,361 DEGs were involved in the regulation of cell processes, accounting for 84.73% of the total annotated genes; 13,296 DEGs were related to biological regulation, representing 61.35% of the total annotated genes; and 12,278 DEGs were associated with the metabolic process, constituting 56.66% of the total annotated genes. Cellular component primarily included cell anatomical entity and intracellular. Among them, 20,341 DEGs were related to cell anatomica entity, and 19,068 DEGs were involved in intracellular, accounting for 93.87% and 87.99% of the total number of annotated genes, respectively. In the molecular function, 12,231 DEGs participated in binding, and 8072 DEGs were involved in catalytic activity, representing 56.44% and 37.25% of the total annotated genes, respectively. Given that the majority of annotated DEGs were associated with biological processes, it was speculated that DEGs played an important role in the growth and metabolism of *P. citrinopileatus*.

#### 3.4.4. KEGG Pathway Analysis

The KEGG database aims to elucidate the metabolic pathways and biochemical reactions of genes, biomolecules and proteins in cells. Through KEGG analysis, the pathways associated with DEGs and their corresponding functions can be described.

**Figure 13:**
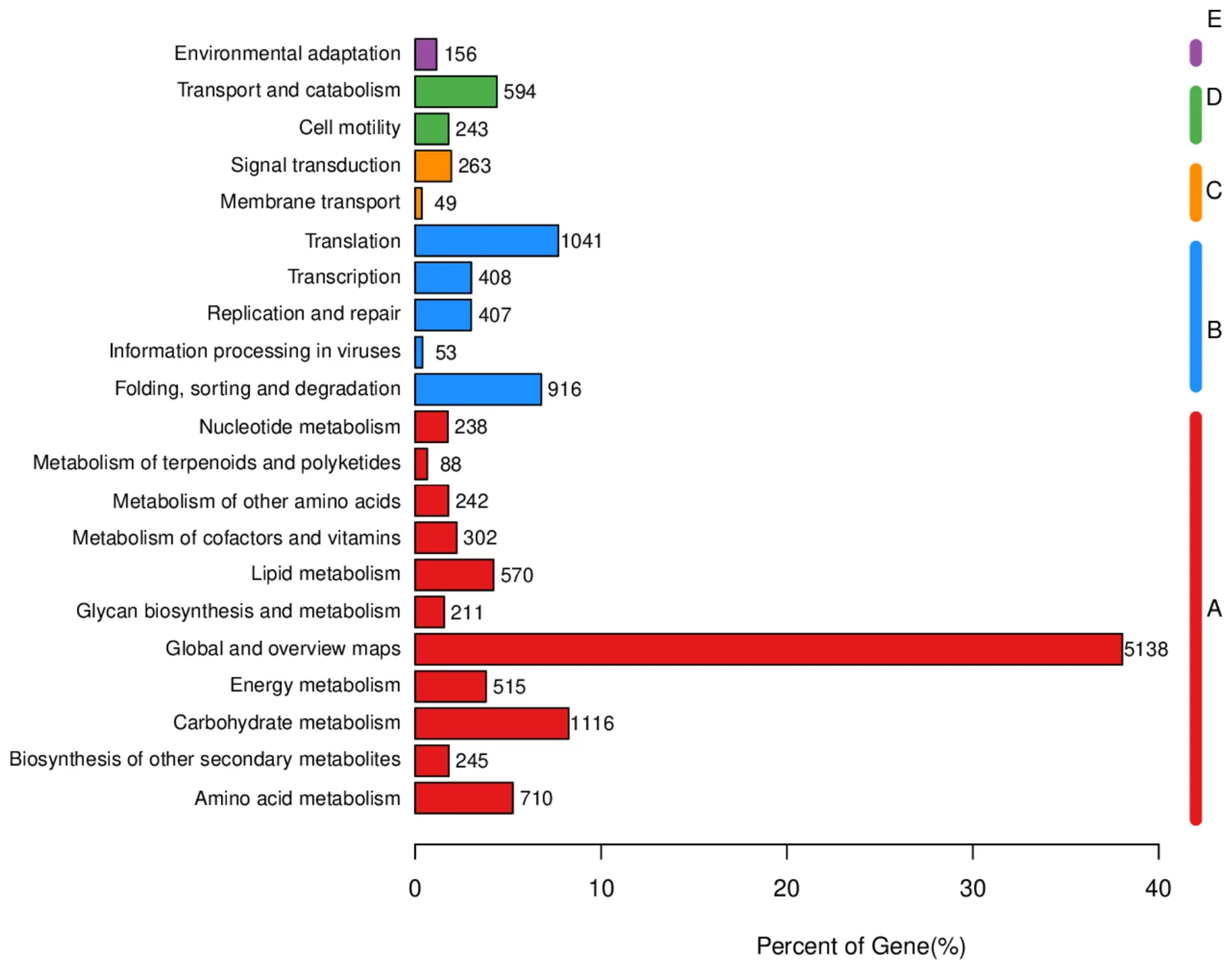
Classification of KEGG pathway. A: metabolism, B: genetic information processing, C: environmental information processing, D: cellular processes, E: organismal systems.

DEGs participated in 132 KEGG pathways. As shown in Figure 13, within the metabolism category, the representative classifications included global and overview maps, along with carbohydrate, amino acid, lipid, and energy metabolism, cofactor and vitamin metabolism, synthesis of other secondary metabolites, etc. In genetic information processing, the main assignments were translation, folding, sorting and degradation, transcription, and replication and repair. Signal transduction and membrane transport were classified under environmental information process, while transport and catabolism, and cell motility were the subclasses in cellular processes. DEGs were also located in pathways related to environmental adaptation in organic systems.

**Figure 14:**
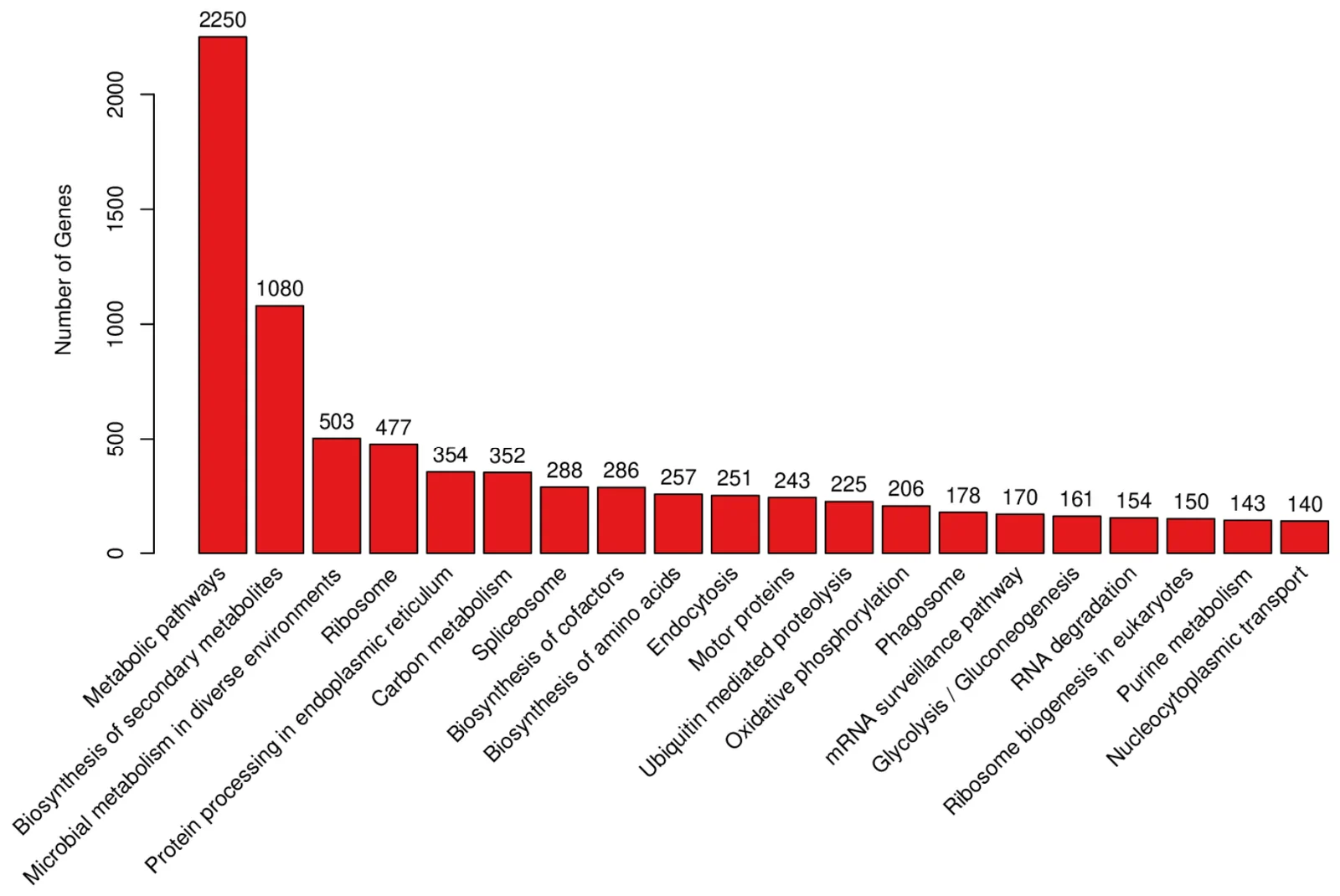
The top 20 KEGG pathways with the largest number of DEGs.

The top 20 KEGG pathways with the highest number of DEGs is shown in Figure 14. The pathway with the largest number of DEGs was metabolic pathways (2250), followed by biosynthesis of secondary metabolites (1080). Other pathways with a large number of DEGs (>250) included microbial metabolism in diverse environments (503), ribosome (477), protein processing in endoplasmic reticulum (354), carbon metabolism (352), spliceosome (288), biosynthesis of cofactors (286), biosynthesis of amino acid (257) and endocytosis (251). The large numbers of DEGs in metabolic pathways and biosynthesis of secondary metabolite indicated LF-AMF might affect the metabolic pathways and metabolites of *P. citrinopileatus*. Furthermore, the involvement of many DEGs in carbon metabolism suggested that LF-AMF might change the type and composition of carbohydrates.

**Figure 15:**
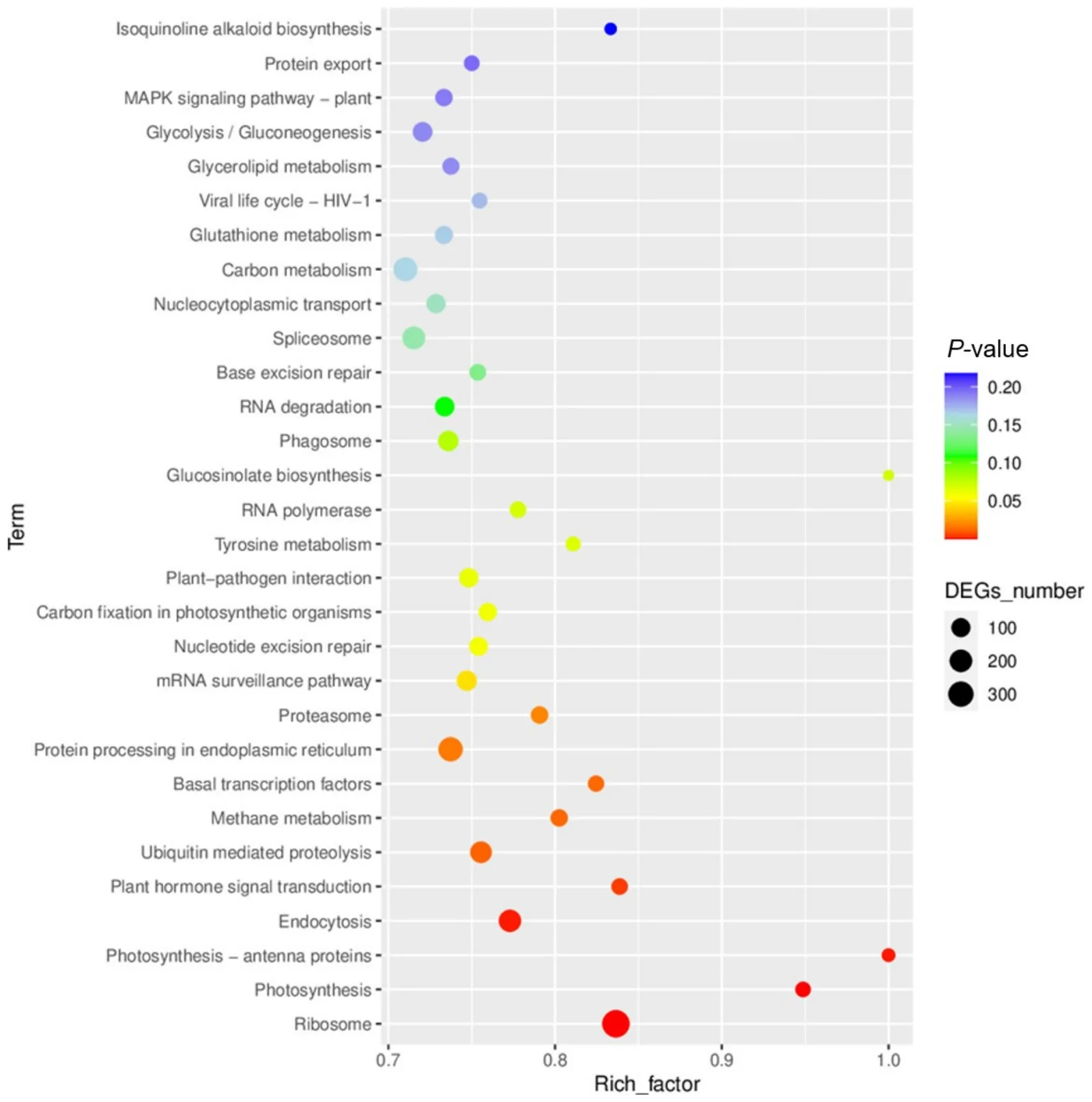
Enrichment of KEGG pathways.

The enrichment of the top 30 KEGG pathway is shown in Figure 15. The extremely significantly enriched pathways (*p* < 0.05) were ribosome, photosynthesis, photosynthesis-antenna proteins, endocytosis, plant hormone signal transduction, ubiquitin mediated proteolysis, methane metabolism, basal transcription factors, protein processing in endoplasmic reticulum, proteasome, and mRNA surveillance pathway. Ribosome and endocytosis were significantly enriched pathways with a large number of DEGs. Eukaryotic ribosomes consist of a small subunit (40S) and a large subunit (60S), which catalyze the synthesis of proteins [40]. Previous studies have shown that ribosomes, which decode genetic information into amino acid sequences and polymerize monomers into proteins, are indispensable for bacterial protein synthesis and cellular viability [41]. It is speculated that LF-AMF also affected protein and amino acid biosynthesis of *P. citrinopileatus*. Endocytosis serves a critical function in the uptake and elimination of macromolecules and particulate matter from the extracellular environment, while also regulating intercellular communication, signal transduction, and homeostasis at both the cellular and organismal levels in eukaryotes [42]. Hyphal growth and morphogenesis in filamentous fungi is also related to endocytosis [43]. LF-AMF changed the uptake of nutrients, and also changed the growth and mycelial morphology of *P. citrinopileatus*, which is in consist with the results from our previous study [44].

#### 3.4.5. Central Carbon and Energy Metabolic Network Analysis

**Figure 16:**
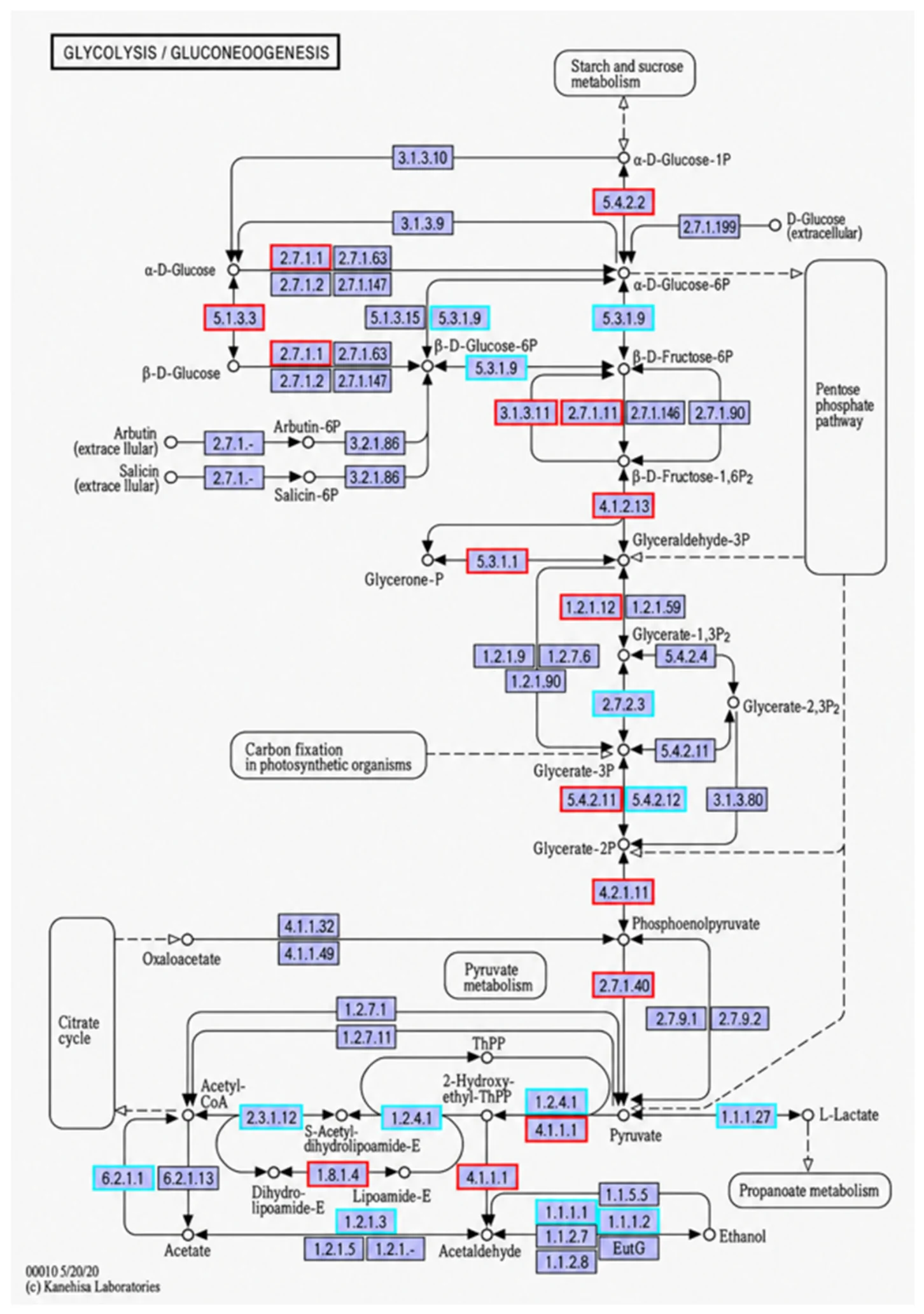
The regulation pathway of Glycolysis/Gliconeogenesis of DEGs.

Glycolysis/gluconeogenesis is a central pathway in microbial monosaccharide metabolism. The regulation pathway of glycolysis/gluconeogenesis involving DEGs is presented in Figure 16. There were 161 DEGs participated in glycolysis/gluconeogenesis. Among these DEGs, the expression levels of hexokinase (2.7.1.1) and 6-phosphofructokinase (2.7.1.11) were up-regulated, while that of glucose-6-phosphate isomerase (5.3.1.9) was down-regulated. Hexokinase is a key rate-limiting enzyme in the glycolysis cycle, converting glucose to glucose-6-phosphate for glycogen synthesis or participation in the pentose phosphate pathway [45]. ATP-dependent 6-phosphofructokinase catalyzes the phosphorylation of fructose-6-phosphate (F6P) to fructose-1, 6-bisphosphate (FBP), an irreversible reaction that is one of the most important steps in glycolysis [46]. In addition, 6-phosphofructokinase is also a key enzyme regulating the glycolytic rate [47]. Glucose-6-phosphate isomerase facilitates the degradation of glucose-6-phosphate [48]. The changes in the expression levels of these genes indicated that LF-AMF promoted the accumulation of fructose-1,6-bisphosphate, thereby accelerating the rate of glycolysis. The expression levels of fructose-1,6-phosphate aldolase (4.1.2.13) that converts fructose-1,6-bisphosphate to glyceraldehyde-3-phosphate and dihydroxyacetone phosphate, glyceraldehyde-3-phosphate dehydrogenase (1.2.1.12) that catalyzes the oxidation (dehydrogenation) and phosphorylation of glyceraldehyde-3-phosphate to produce 1,3-diphosphoglycerate, and 2,3-bisphosphoglycerate-dependent phosphoglycerate mutase (5.4.2.11) which may lead to an increase in the amount of fructose phosphate synthesis were up-regulated. Fructose phosphate plays an important role in glycolysis and gluconeogenesis, not only related to the final product, but also essential for the synthesis of nucleotide sugars, which can participate in the synthesis of EPS. In the liquid fermentation of *P. citrinopileatus*, the improved yield of EPS due to LF-AMF may be attributed to the accumulation of fructose phosphate. The expression level of phosphoglucomutase (5.4.2.2) was also up-regulated. Phosphoglucomutase catalyzes the reversible interconversion between glucose-6-phosphate and glucose-1-phosphate, and the latter serves as a core precursor for nucleotide sugar synthesis, which are essential activated substrates for EPS biosynthesis.

**Figure 17:**
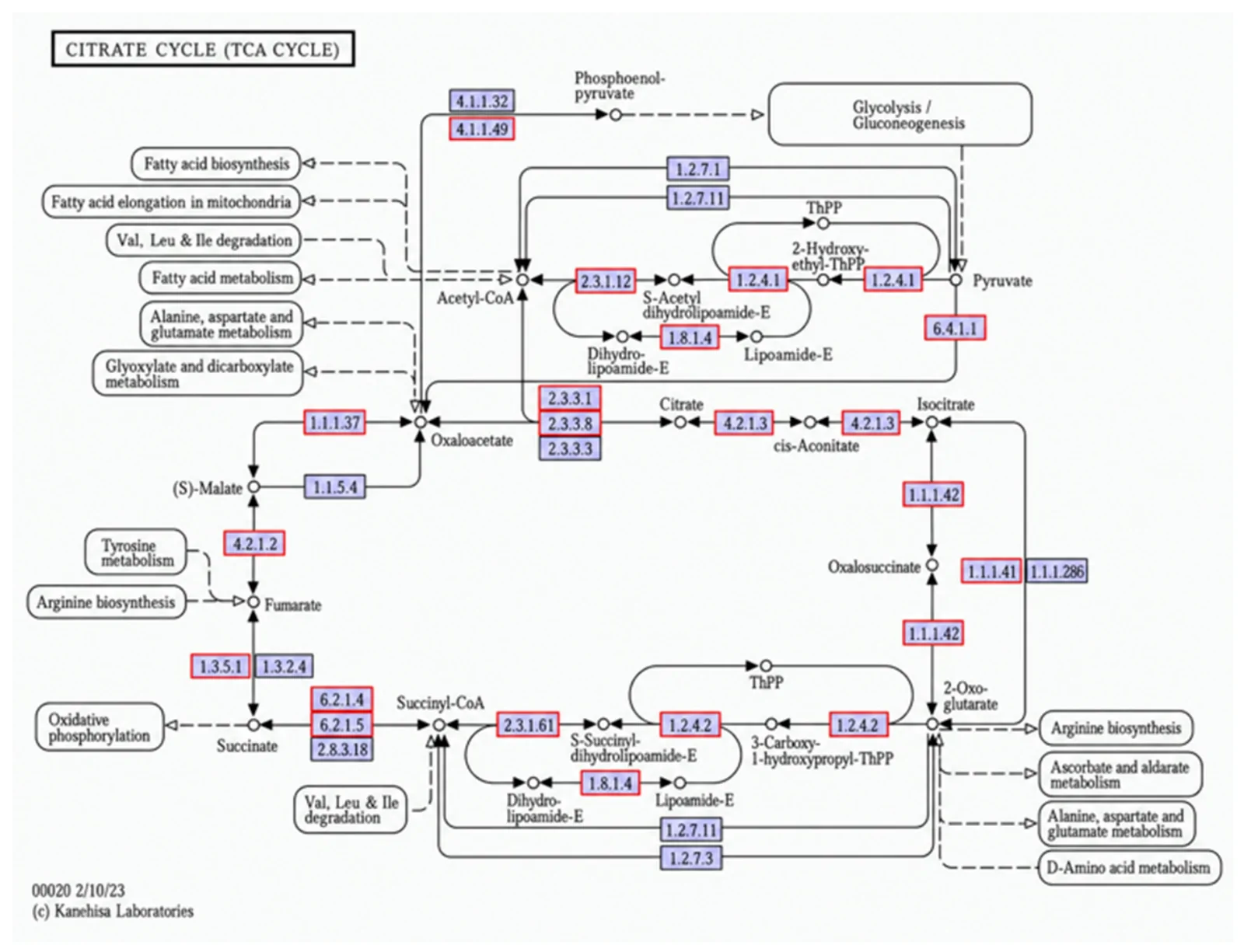
The regulation pathway of TCA cycle of DEGs.

The energy metabolism of *P. citrinopileatus* mainly depends on the tricarboxylic acid (TCA) cycle, which plays an important role in the aerobic metabolic pathway of organisms. As an efficient way for organisms to obtain energy, the TCA cycle also affects the synthesis of metabolites and the metabolism of carbohydrates, lipids and amino acids. The regulation pathway of TCA cycle involving DEGs is shown in Figure 17. The gene expression levels of citrate synthase (2.3.3.1), isocitrate dehydrogenase (1.1.1.42) and α-ketoglutarate dehydrogenase (1.8.1.4) in the TCA cycle were up-regulated. Consistent with the finding that external intervention reshapes organismal carbon and energy metabolism [49], LF-AMF may enhance the fermentation efficiency by improving the energy metabolism of *P. citrinopileatus*, and changes in energy level may also affect the synthesis of products. Citrate synthase is an allosteric enzyme in the TCA cycle that regulates the first step of the TCA cycle and promotes the synthesis of citrate coenzyme A. Isocitrate dehydrogenase acts as a rate-limiting enzyme in the TCA cycle, rapidly decarboxylating isocitrate to produce α-ketoglutarate. α-ketoglutarate dehydrogenase can convert α-ketoglutarate to succinyl coenzyme A, NADH+H^+^ and CO_2_.

## 4. Conclusions

In this study, we investigated the regulatory effects of low-frequency alternating magnetic field (LF-AMF) on *P. citrinopileatus* fermentation, with a focus on the structural and functional alterations of exopolysaccharide (EPS) and the underlying metabolic regulatory mechanism. LF-AMF-assisted fermentation changed the type and structural characteristics of EPS secreted by *P. citrinopileatus*, such as its monosaccharide composition and molecular weight. LF-AMF-assisted fermentation improved the thermal stability of EPS, modulated its flocculation activity, and maintained emulsifying activity comparable to that of normal fermentation. Transcriptome analysis revealed that the expression levels of genes involved in glycolysis and the TCA cycle were affected by LF-AMF. Those changes influenced the biosynthesis of EPS, resulting in the alterations in its structural and physicochemical properties. Future research should focus on the biological activities of EPS produced under LF-AMF, including hypoglycemic, anti-tumor, anti-inflammatory and other functions, thereby facilitating its broader application and enhancing its economic value.

## Figures and Tables

**Table 1:** Statistics of sequencing reads and base comparison.

Sample	Total Raw Reads (Mb)	Total Clean Reads (Mb)	Total Raw Bases (Gb)	Total Clean Bases (Gb)	Clean Reads Q20 (%)	Clean Reads Q30 (%)
Control	27.70 ± 0.9	25.17 ± 3.18	4.15 ± 0.15	3.67 ± 0.47	98.66 ± 0.73	98.05 ± 0.17
Sample	27.02 ± 1.85	25.97 ± 1.55	4.05 ± 0.28	3.81 ± 0.22	99.26 ± 0.17	97.82 ± 0.40

**Table 2:** Reads alignment results against the reference genome.

Sample	Total Clean Reads (Mb)	Mapped Reads	Mapping Rate (%)
Control	25.17 ± 3.18	22.48 ± 2.85	89.30 ± 2.28
Sample	25.97 ± 1.55	24.11 ± 1.38	92.86 ± 1.00

## Data Availability

The original contributions presented in this study are included in the article. Further inquiries can be directed to the corresponding author.
